# Differential Item Functioning of the Psychological Domain of the Menopause Rating Scale

**DOI:** 10.1155/2016/8790691

**Published:** 2016-10-26

**Authors:** Alvaro Monterrosa-Castro, Katherin Portela-Buelvas, Heidi C. Oviedo, Edwin Herazo, Adalberto Campo-Arias

**Affiliations:** ^1^Grupo de Investigación Salud de la Mujer, Facultad de Medicina, Universidad de Cartagena, Cartagena, Colombia; ^2^Grupo de Investigación del Comportamiento Humano, Instituto de Investigación del Comportamiento Humano, Bogotá, Colombia

## Abstract

*Introduction.* Quality of life could be quantified with the Menopause Rating Scale (MRS), which evaluates the severity of somatic, psychological, and urogenital symptoms in menopause. However, differential item functioning (DIF) analysis has not been applied previously.* Objective*. To establish the DIF of the psychological domain of the MRS in Colombian women.* Methods*. 4,009 women aged between 40 and 59 years, who participated in the CAVIMEC (Calidad de Vida en la Menopausia y Etnias Colombianas) project, were included. Average age was 49.0 ± 5.9 years. Women were classified in mestizo, Afro-Colombian, and indigenous. The results were presented as averages and standard deviation (*X* ± SD). A *p* value <0.001 was considered statistically significant.* Results*. In mestizo women, the highest *X* ± SD were obtained in physical and mental exhaustion (PME) (0.86 ± 0.93) and the lowest ones in anxiety (0.44 ± 0.79). In Afro-Colombian women, an average score of 0.99 ± 1.07 for PME and 0.63 ± 0.88 for anxiety was gotten. Indigenous women obtained an increased average score for PME (1.33 ± 0.93). The lowest score was evidenced in depressive mood (0.50 ± 0.81), which is different from other Colombian women (*p* < 0.001).* Conclusions*. The psychological items of the MRS show differential functioning according to the ethnic group, which may induce systematic error in the measurement of the construct.

## 1. Introduction

Quality of life in menopause could be quantified with the use of instruments as the Menopause Rating Scale (MRS); it is a scale that measures common symptoms attributed to menopause [1]. However, measurements could be biased if the scale does not have high validity [2].

Bias measurements are presented when a differential response pattern is observed, according to an external characteristic of the instrument (age, education, ethnic group, etc.), which does not have any expected or important relationship with the construct [3]. Differential functioning represents a big problem because it affects scale validity [2].

Recently, the item response theory (IRT) has allowed identifying systematically differentiated or biased patterns because of an external condition of the construct that the scale quantifies using different techniques. IRT allows the validation of scales for clinical assessments or epidemiological studies. The best health measurement scale is one that does not show item bias [4].

Based on the classic theory, factor analysis and internal consistency of MRS have been studied in different contexts and populations [5]. However, DIF of the MRS is unknown. In the present study, the DIF analysis for the psychological domain of MRS is assessed among three ethnic groups of Colombian women.

The MRS consists of three domains of somatic, psychological, and urogenital symptoms [1]. MRS has been validated around the world, was originally written in German, and was translated into Spanish, French, English, Portuguese, Swedish, Turkish, and Indonesians dialects [6]. The translation process tries to eliminate possible systematic mistakes due to problems in the semantic equivalences of the words used in the different idioms [7].

The mean aim of this study was to analyze the DIF for the psychological domain of the MRS, according to the ethnic group, in a big sample of Colombian women.

## 2. Methods

The present study was carried out with a total of 4,009 women, who participated in the CAVIMEC research project (Calidad de Vida en la Menopausia y Etnias Colombianas). Women were aged between 40 and 59 years (49.0 ± 5.9). According to ethnic, 1,810 women (45.1%) were mestizo; 1,285 (32.1%) Afro-Colombian; and 914 (22.8%) from different indigenous populations resident in three settlements (Wayuú in La Guajira, Zenu in the State of Cordoba, and different native groups of Amazonas).

The MRS is a self-applied scale. It is compounded of 11 items; four of them make part of the psychological domain. The domain explores depressive symptoms (feeling down, sad, on the verge of tears, lack of drive, and mood swings), irritability (feeling nervous, inner tension, and feeling aggressive), anxiety (inner restlessness, feeling panicky), and the physical and mental exhaustion (general decrease in performance, impaired memory, decrease in concentration, and forgetfulness). Scale offers five options of answers, absent, mild, moderate, severe, and very severe (reference).

All participants completed the scale by themselves. Nevertheless, for women with limitations for reading in Spanish a nurse with skills in the community language helped them to understand the form in their native dialect.

To know the performance of items, according to the ethnic groups, two tests were employed: the analysis of variance (ANOVA) test and Spearman's correlations (*r*_*s*_). In the first test, *p* values < 0.001 indicated differential functioning [8] and with Spearman's correlations (*r*_*s*_) with its confidence interval of 95%, *r*_*s*_ = 0 indicated absence of a differential response pattern by ethnic group [9]. For calculations, zero [0], one [1], and [2] two values were assigned for mestizo, Afro-Colombian, and indigenous women, respectively. Finally, the classic reliability tests to quantify internal consistency were computed, Cronbach's alpha (*α*) [10] and McDonald's omega (*ω*) [11].

## 3. Results

Table 1 indicates all the psychological items of the MRS and shows a differential functioning by ethnic group. Mestizo women had the highest score in depressive mood, Afro-Colombian women had the highest score in anxiety, and indigenous women showed the highest score in irritability and physical and mental exhaustion.

Similarly, by ethnic group, the correlations (*r*_*s*_) showed a differential functioning. In Table 2, it is observed that all values were different to zero, with a confidence interval of 95%, especially for irritability and mental and physical exhaustion.

The psychological domain showed high internal consistency in the three ethnic groups, *α* = 0.78 and *ω* = 0.79 for mestizo women; *α* = 0.81 and *ω* = 0.83 for Afro-Colombian participants; and *α* = 0.75 and *ω* = 0.77 in indigenous women.

## 4. Discussion

In the present study, we observed a differential performance of the items of psychological domain of MRS, using two different statistical tests, in three different ethnic groups of Colombian women.

The DIF is a strategy to study the construct validity of the measurement instrument. DIF is based on the item response theory (IRT) which is highly used in different knowledge areas and recently introduced in the assessment of the performance of some health measures [3].

The approximation is based on the IRT and has the following advantage: compared with those assessments based on the classic theory of the scales, it is able to identify bias in the response of each item of the scale [4, 12].

The DIF of the psychological items is not completely new. Some important variations have been observed in the frequency of menopausal symptoms that are manifested by women: changes that could be mediated by physiological differences between the ethnic groups, the sociocultural environment, or the interaction between these variables [13].

In spite of the process of formal translation of the MRS from German into Spanish it is possible that the DIF by ethnic group observed in the current analysis could be attributed to or explained by some linguistic differences between mestizo and Afro-Colombian women, who speak and read habitual Spanish and who were not considered at the time of the scale application. These adjustments are always necessary given the semantic variations that are present between different population groups [14]. In addition, other aspects should be considered, such as the possible effect produced by the application of the scale by another person and its translation to the language of the different indigenous group who participated [15].

Finally, the observed bias could be hidden in the construction and own conceptualization of the scale and in the construct named “menopause,” which is always expected to have similar behavior in all the contexts [2].

Without doubt, these findings suggest the need to do some adjustments in the writing of the items in order to avoid mistakes in the interpretation and, therefore, in the answer of the items psychological domain of the MRS [7, 14]. And in the same way, some studies showed significant differences in the symptoms frequency according to the sociocultural context of the participants [16–19]; those differences might indicate the need of reviewing the group of typical symptoms attributed to menopause, because it implies, since the psychometrics view, a refinement of the underlying theoretical construct [20]. And also, it invites to question the internal validity and the conclusions of all the studies carried out with the MRS in order to quantify the symptoms in all the domains, mainly in the psychological domain [2, 3].

It is concluded that the items of the psychological domain of the MRS show differential functioning according to the ethnic group. The finding could produce a systematic mistake in the measurement of the construct or lack of adjustments to the linguistic and semantic distinctive features of each Colombian ethnic group. A careful assessment of this dimension of the scale is required.

## Figures and Tables

**Table 1:** Average for psychological items of the MRS according to the ethnic group.

Item	Mestizo	Afro-Colombian	Indigenous	Total
Depressive mood	0.76 (0.97)	0.74 (0.93)	0.50 (0.81)	0.70 (0.93)
Irritability	0.56 (0.85)	0.67 (0.88)	0.85 (0.74)	0.66 (0.84)
Anxiety	0.44 (0.79)	0.63 (0.88)	0.55 (0.80)	0.53 (0.83)
Physical and mental exhaustion	0.86 (0.93)	0.99 (1.07)	1.33 (0.93)	1.01 (0.99)

*p* < 0.001 (which one?).

**Table 2:** Spearman correlation (*r*_*s*_) and confidence interval of 95% according to the ethnic group.

Item	*r*_*s*_ (mestizo < Afro-Colombian < indigenous)
Depressive mood	−0.091 −(0.060–0.122)
Irritability	0.177 (0.147–0.207)
Anxiety	0.092 (0.061–0.123)
Physical and mental exhaustion	0.182 (0.152–0.212)
